# Patterns of healthcare utilization according to health equity determinants during the first year of the pandemic at Johns Hopkins Medicine

**DOI:** 10.1093/jamiaopen/ooae093

**Published:** 2024-10-07

**Authors:** Kai-Wen K Yang, Ilia Rattsev, Zoljargal Lkhagvajav, Natalie Flaks-Manov, Kevin Gorman, Jeremy Aaron Epstein, Ciprian M Crainiceanu, Casey O Taylor

**Affiliations:** Johns Hopkins Whiting School of Engineering, Institute for Computational Medicine, Baltimore, MD 21218, United States; Johns Hopkins Whiting School of Engineering, Institute for Computational Medicine, Baltimore, MD 21218, United States; Department of Biomedical Engineering, Johns Hopkins University, Baltimore, MD 21218, United States; Johns Hopkins Whiting School of Engineering, Institute for Computational Medicine, Baltimore, MD 21218, United States; Johns Hopkins Whiting School of Engineering, Institute for Computational Medicine, Baltimore, MD 21218, United States; Johns Hopkins Whiting School of Engineering, Institute for Computational Medicine, Baltimore, MD 21218, United States; Division of Hospital Medicine, Johns Hopkins Hospital, Baltimore, MD 21224, United States; Department of Biostatistics, Johns Hopkins Bloomberg School of Public Health, Baltimore, MD 21205, United States; Johns Hopkins Whiting School of Engineering, Institute for Computational Medicine, Baltimore, MD 21218, United States; Department of Biomedical Engineering, Johns Hopkins University, Baltimore, MD 21218, United States; Division of General Internal Medicine, Department of Medicine, Johns Hopkins University School of Medicine, Baltimore, MD 21231, United States

**Keywords:** telemedicine, COVID-19, health equity, social determinants of health, patient acceptance of health care

## Abstract

**Objectives:**

Rapid telehealth adoption happened at the onset of the coronavirus disease 2019 (COVID-19) pandemic, resulting in a move from *in-person predominant* to *telehealth predominant* care delivery. Later, in person visits rebounded with telehealth options remaining. This study aimed to assess differences in healthcare utilization during this changing landscape in terms of health equity determinants.

**Materials and Methods:**

This was an observational cohort study of Johns Hopkins Medicine (JHM) patients. We analyzed utilization of video, telephone, and in-person patient-provider visits by eligible patients between March 16, 2019 and December 31, 2020. Percent changes in average weekly patient-provider visits from *pre-pandemic* (March 16, 2019-June 30, 2019) to *early 2020 pandemic* (March 16, 2020-June 30, 2020) and from *pre-pandemic* (July 1, 2019-December 31, 2019) to *late 2020 pandemic* (July 1, 2020-December 31, 2020). We used a quantile cut off technique to describe disproportionately smaller or greater drops in visits during the first year of the pandemic among health equity determinant groups and according to visit specialty, when compared to the total population.

**Results:**

There was a 39% drop in patient-provider visits from the pre-pandemic to the early 2020 pandemic period, and a 24% drop from pre-pandemic to the late 2020 pandemic period. We discovered 21 groups according to health equity determinates and visit departments with patterns of disproportionately smaller or greater drops in visits during the first year of the pandemic, when compared to the total population: *Pattern 1***—**smaller drop in visits early and late 2020 (age 45-64, Medicare insurance, high poverty and high unemployment; mental health and medical specialty visits –*P* < .001); *Pattern 2***—**greater drop in visits early 2020 only (age 65-84; OB/GYN and surgical specialty visits—*P* < .001); *Pattern 3***—**greater drop in visits early and late 2020 (age 0-5, age 6-17, age 85+, Asian race, Hispanic or Latino ethnicity, private insurance—*P* < .001); and *Pattern 4*—smaller drop in visits in early 2020 when compared to late 2020. The age 18-44 group showed a smaller drop in visits early 2020 and then visit levels similar to the total population late 2020. Primary care visits were similar to the total population early 2020 and then a smaller drop in visits late 2020 (*P* < .001).

**Discussion:**

Our study provides evidence of health equity determinant groups having disproportionally smaller or greater drops in visits during the first year of the pandemic. The observed differences may have been influenced by changing telehealth offerings during the first year of the pandemic. Groups with disproportionately smaller drops in visits early 2020 (*Pattern #1* and age 18-44 group in *Pattern #4*), suggests more success with adopting telehealth among those groups. Whereas groups with disproportionately greater drops in visits early 2020 (*Pattern #2* and *Pattern #3*), suggests less success with telehealth adoption. For *Pattern #4*, more clarification is needed on how changes in telehealth offerings contributed to the downward trend in visits observed from early to late 2020.

**Conclusion:**

We describe 4 main patterns to characterize groups with disproportionately smaller or greater drops in visits during the first year of the pandemic. While this work did not specifically study vulnerable populations, these patterns set the stage for further studies of such groups.

## Background and significance

After the first cases of the COVID-19 pandemic (March 2020)^1^ and the Declaration of State of Emergency,^2^ most providers made a rapid shift to telehealth with in-person visits occurring at much lower rates. Johns Hopkins Medicine (JHM) has had an office of telemedicine since 2016, but due to billing and regulatory barriers, uptake remained low. By February 2020, we had nearly 800 documented ambulatory telemedicine visits, around 70/month in the months before March 2020. Thus, this early 2020 pandemic period represented a move from largely “in-person predominant” care delivery to “*Telehealth Predominant*” care delivery. The number of in person visits began to rebound around June 2020^3^ with telehealth options remaining. As such, we refer to this as the late 2020 pandemic period as a “*In-person & Telehealth*” period. Notably, during this time, the number of telehealth visits began to decline in some, but not all, areas of healthcare.^4^ These changes in healthcare telehealth offerings were accompanied by corresponding changes in insurance coverage of video-based telemedicine, which are likely to incentivize many providers to continue to devote institutional resources to telehealth.

For many vulnerable populations, telehealth services hold promise to reduce barriers to care due to transportation and childcare needs, and the expanded payment policies broadened access to such services.^5^^,^^6^ Strategies for offering telehealth, however, may increase the existence of and create new health inequities by disproportionately improving healthcare access for some,^7–11^ while leaving vulnerable patients behind.^12^ Several studies provide evidence of disparities in telehealth use during the pandemic,^13–17^ but more work is needed to understand healthcare utilization among groups according to health equity determinants (eg, living in an area of high poverty) during the first year of the pandemic that includes a period of “telehealth predominant” care delivery.

In addition, prior studies of trends in healthcare utilization during the pandemic compare pre-pandemic and pandemic healthcare utilization,^18–21^ and have compared healthcare utilization late 2020 and 2021 during the pandemic.^22^^,^^23^ There is a gap, however, in studies of healthcare utilization during the full first year of the pandemic that includes a period when in-person visits were just beginning to be reintroduced (the late 2020 pandemic period). This study covers this critical period when low and medium risk activities were being reopened in Maryland in May and June 2020.^24^ While in-person healthcare visits were allowed before then, fear of contracting COVID-19 and reallocation of medical resources to fight the outbreak prevented many individuals from seeking non-essential medical care early in the pandemic.^25^

We examined the healthcare utilization among groups defined according to health equity determinants and visit specialties over the first year of the pandemic. Main contributions of this paper are: (1) characterizing patient-provider visit utilization (video, telephone, and in-person) during time periods the first year of the pandemic when we experienced changes in telehealth offerings; (2) identifying patterns of patient-provider visits during the first year of the pandemic in a diverse population in terms of a range of health equity determinants (demographics, neighborhood-level social determinants of health (SDOH), insurance status); and (3) Identifying and discussing plausible reasons for disproportionately smaller or greater drops in visits for some health equity determinant groups and visit specialties, relative to the total population.

## Objectives

The objectives of this study were to: (1) Characterize patient-provider visit utilization during the early 2020 (*Telehealth Predominant*) pandemic period and the late 2020 (*In-person & Telehealth*) pandemic period relative to a year prior to the pandemic during those time periods; (2) Identify the patterns in patient-provider visit utilization by comparing visits during the early 2020 pandemic period and the late 2020 pandemic period, to a year prior to the pandemic during those time periods; and (3) Assess patient-provider visit utilization patterns for disproportionately smaller or greater drops in visits when compared to the total population in terms of health equity determinants and visit specialties.

## Materials and methods

This was an observational cohort study of JHM patients (Johns Hopkins Hospital, Johns Hopkins Bayview Medical Center, Johns Hopkins Community Physicians). To avoid non- or low-users of JHM facilities, we extracted the encounter data of only those patients who had visited a primary care provider at least once in a 12-month period prior to March 16, 2020. To better understand differences in healthcare utilization during periods of different in-person and telehealth offerings at JHM, we assessed video, telephone and in-person visits during two pandemic periods:

- *Early 2020 pandemic (Telehealth Predominant)*: March 16, 2020-June 30, 2020.
- *Late 2020 pandemic (In-person & Telehealth)*: July 1, 2020-December 31, 2020.

March 16, 2020 was chosen as the starting point of the early 2020 pandemic (*Telehealth Predominant*) period because the Governor of Maryland prohibited public gatherings from this day.^26^ We selected June 30, 2020 to be the end of the early 2020 period because Stage 2 reopening began in June 2020^27^ and in-person visits were reintroduced. To adjust for seasonal fluctuations in healthcare utilization, we compared visit utilization during the early 2020 pandemic and late 2020 pandemic periods to corresponding pre-pandemic (*In-person Predominant*) periods in 2019.

Electronic health record (EHR) data was used to measure patient-provider visit utilization, including video, telephone and in person visits. Patient-provider visits were limited to those with a practitioner with an M.D. (Doctor of Medicine) or D.O. (Doctor of Osteopathic Medicine) degree. In addition, only those visits with appointment status recorded as complete were included in the dataset. Drawing from local knowledge of clinical documentation practices at JHM, co-author JE selected outpatient visit encounter types (that included video, telephone and in-person visit types, **Table S1**), provider types, and provider specialties to be included. We also excluded encounters such as lab testing, imaging exams, and vaccination records. We considered visit utilization among patient groups according to three types of health equity determinants: demographic characteristics (age, gender, race, and ethnicity), neighborhood-level SDOH, and insurance status. The SDOH variables included 4 variables to characterize the economic status, social and neighborhood characteristics, and housing and transportation availability at the census-tract level: percent living in poverty, percent unemployed, percent with no high school diploma, and percent with no vehicle. To understand such differences in utilization across hospital departments, we also conducted the analysis by specialty groups. The demographic factors were obtained for each patient and insurance and specialty for each encounter from EHR data. The binary low-high SDOH characteristics were assigned to patients based on their census track 11-digit Federal Information Processing System (FIPS) code documented in the EHR with the SDOH data published in ref.^28^ We define neighborhoods with >40% persons below poverty estimates as high poverty, while >20% civilian (age 16+) unemployed, >25% persons with no high school diploma (age 25+), and >20% households with no vehicle available as high unemployment, low education, and low vehicle possession, respectively. The poverty thresholds used were in accordance with the Census Bureau,^29^ which uses a set of dollar value thresholds that vary by family size and composition to determine who is in poverty. This study was approved by the Institutional Review Board of Johns Hopkins University (protocol code IRB00245964, approved 5/9/2020).

To quantify the patient-provider visit utilization, we calculated the average weekly numbers of visits (video, telephone and in-person) among our cohort across the study periods. Absolute and percent changes in average weekly visits from the *pre-pandemic (In-person-Predominant) period* to the *early (Telehealth-Predominant)* and *late pandemic (In-person & Telehealth) periods* were obtained among the total population and within health equity determinant groups and according to visit specialty. Furthermore, the percent change in average weekly patient-provider visits among the groups were compared to the percent change in the total population using the unpaired *z*-test for proportions. Conditional on the time period, the observations were assumed to be independent. The statistical significance criterion was *P* < .05.

To focus our discussion, we discover patterns of disproportionately smaller or greater drops in visits observed in health equity determinant groups and according to visit specialty, relative to the total population. We used a quantile cut off technique that involved separating the groups into those that had percentage changes less than and more the average observed for the total population, and obtained the quantile loss or gain for each group. This approach resulted in 8 quantile groups for each of the early and late 2020 pandemic periods (below −Q3, −Q3 to −Q2, −Q2 to −Q1, −Q1 to total, total to +Q1, +Q1 to +Q2, +Q2 to +Q3, and above +Q). We use these quantiles to define patterns for groups with drops in visits that appeared to be substantially smaller or greater than the total population during early or late 2020 pandemic periods (eg, groups with smaller drops in visits throughout the first year of the pandemic). To help visualize groups with large differences, we plot early 2020 pandemic quantiles on the *x*-axis and late 2020 pandemic quantiles on the *y*-axis. Groups that visually fall within the second quantiles (−Q_2_ to +Q_2_) around the total for both early and late 2020 pandemic periods were not described further due to their close proximity to the total average during the first year of the pandemic.

## Results

### Patient population

The patient-provider visits were analyzed for a cohort of 89 371 patients. A total of 659 448 patient-provider visits were completed during the study period. Descriptive statistics of the cohort are presented in Table 1. There were more females than males (58% vs 42%), and most represented age groups included patients aged 45-64 and 65-84 (32% and 29%, respectively). The cohort had a larger proportion of individuals who identified as White (55%) and a smaller proportion of individuals who identified as Black (31%). The majority of the patients were Not Hispanic (92%), lived in neighborhoods with lower rates of poverty (≤40% in poverty) (98%), lower rates of unemployment (≤20% unemployed) (94%), had higher levels of education (≤25% with no high school diploma) (93%), and had higher vehicle possession rates (≤20% with no vehicle) (83%). There were more encounters associated with private insurance (38%), followed by Medicare (33%), while most encounters were of primary care (65%) specialty, followed by surgical (14%) and medical (11%) specialties. The racial composition of this cohort is similar to that of the patient population being seen at the Johns Hopkins Health System in Maryland^30^ and the Maryland Census.^31^ Thus, we believe our study results are representative of the overall population of the Maryland area.

**Table 1.** Patient health equity determinates and visit specialties, and changes in the average weekly number of patient-provider visits during early 2020 pandemic and late 2020 pandemic periods as compared to pre-pandemic.

	Number of patients (% of the total population)	Total number of visits within the study period (% of total visits)	Early 2020 pandemic (March 16th-June 30th)				Late 2020 pandemic (July 1-December 31)			
			AWV (2019) *n *=* *67 813)	AWV (2020) *n *=* *47 422	Relative % diff.	*P*-val.	AWV (2019) *n *=* *83 311	AWV (2020) *n *=* *67 121	Relative % diff.	*P*-val.
Total	89 371 (100%)	659 448 (100%)	9654	5899	−38.89%		9324	7065	−24.23%	
Age										
0-5	8644 (10%)	53 258 (8%)	982	358	−63.50%	****	882	392	−55.56%	****
6-17	6907 (8%)	30 609 (5%)	469	212	−54.82%	****	483	300	−37.82%	****
18-44	16 420 (18%)	102 974 (16%)	1457	1015	−30.29%	****	1440	1093	−24.12%	0.656
45-64	28 788 (32%)	220 064 (33%)	3055	2118	−30.67%	****	2994	2484	−17.03%	****
65-84	25 509 (29%)	225 109 (34%)	3269	1959	−40.07%	****	3122	2519	−19.33%	****
85+	3103 (3%)	27 434 (4%)	420	235	−44.00%	****	400	275	−31.18%	****
Gender										
Female	51 938 (58%)	399 510 (61%)	5767	3644	−36.81%	****	5576	4358	−21.84%	****
Male	37 424 (42%)	259 899 (39%)	3885	2254	−41.97%	****	3747	2706	−27.78%	****
Race										
White	49 390 (55%)	347 850 (53%)	5086	3089	−39.27%	0.081	4952	3709	−25.11%	****
Black	27 696 (31%)	233 961 (35%)	3381	2188	−35.26%	****	3211	2573	−19.86%	****
Asian	3936 (4%)	24 194 (4%)	371	185	−50.06%	****	364	244	−33.03%	****
Multiracial	1211 (1%)	8121 (1%)	126	69	−45.28%	****	122	77	−37.12%	****
Other Race	7138 (8%)	45 322 (7%)	689	367	−46.69%	****	672	461	−31.47%	****
Ethnicity										
Not Hispanic or Latino	81 904 (92%)	612 679 (93%)	8951	5517	−38.36%	***	8629	6587	−23.67%	****
Hispanic or Latino	4982 (6%)	31 121 (5%)	469	248	−47.13%	****	472	310	−34.30%	****
Unknown Ethnicity	2485 (3%)	15 648 (2%)	233	134	−42.45%	****	222	167	−24.49%	0.641
Neighborhood-Level SDOH										
Low Poverty	87 725 (98%)	642 759 (97%)	9411	5738	−39.03%	0.430	9097	6883	−24.34%	0.366
High Poverty	1646 (2%)	16 689 (3%)	242	161	−33.31%	****	226	181	−19.74%	****
Low Unemployment	84 400 (94%)	609 160 (92%)	8925	5415	−39.33%	*	8649	6506	−24.77%	****
High Unemployment	4971 (6%)	50 288 (8%)	729	484	−33.53%	****	675	558	−17.23%	****
High Education	82 812 (93%)	596 492 (90%)	8739	5293	−39.43%	***	8466	6379	−24.65%	****
Low Education	6559 (7%)	62 956 (10%)	915	606	−33.74%	****	857	685	−20.10%	****
High Vehicle Possession	73 883 (83%)	514 712 (78%)	7550	4531	−39.98%	****	7341	5485	−25.29%	****
Low Vehicle Possession	15 488 (17%)	144 736 (22%)	2104	1368	−34.96%	****	1983	1580	−20.31%	****
Insurance										
Medicare		214 841 (33%)	2985	2029	−32.02%	****	2900	2468	−14.90%	****
Medicaid		84 869 (13%)	1259	792	−37.09%	****	1200	880	−26.69%	****
Private		251 139 (38%)	3776	2190	−42.00%	****	3641	2576	−29.25%	****
Other		105 706 (16%)	1580	880	−44.32%	****	1528	1117	−26.90%	****
Specialty										
Primary care		429 719 (65%)	6346	4044	−36.26%	****	6161	4371	−29.04%	****
Surgical speciality		89 152 (14%)	1387	483	−65.18%	****	1322	1027	−22.28%	****
Medical speciality^a^		73 793 (11%)	1020	717	−29.66%	****	981	854	−12.92%	****
Mental health		22 852 (3%)	271	325	19.85%	****	229	305	32.74%	****
OB/GYN		14 402 (2%)	209	93	−55.12%	****	207	171	−17.37%	****
Other speciality^b^		29 530 (4%)	420	235	−44.00%	****	422	334	−20.73%	****

(1)

* *P*-value < .05,

*** *P*-value < .005,

**** *P*-value < .001. (2) AWV = Average weekly visits (*n* represents the number of patients having at least one visit during the respective period). (3) Groups with <20 cell entries are not shown (Gender: other, unknown, nonbinary. Insurance: self-pay).

a Includes provider specialty visits to: addiction medicine, allergy and immunology, cardiac electrophysiology, cardiology, endocrinology, gastroenterology, geriatric medicine, gerontology, hematology, hematology and oncology, hepatology, immunology, infectious disease, interventional cardiology, medical genetics, medical oncology, nephrology, oncology, palliative care, pediatric allergy and immunology, pediatric cardiology, pediatric endocrinology, pediatric gastroenterology, pediatric hematology and oncology, pediatric oncology, pediatric pulmonology, pulmonary disease, radiation oncology, rheumatology, sleep medicine.

b Includes provider specialty visits to: advanced heart failure and transplant, critical care medicine, dermatology, emergency medicine, genetics, integrative medicine, interventional radiology, neuro-ophthalmology, neurology, neuromuscular medicine, occupational medicine, pain medicine, pediatric anesthesiology, pediatric critical care medicine, pediatric dermatology, pediatric neurology, physical medicine and rehabilitation, rehabilitation, transplant, vascular medicine, vascular neurology.

### Main results

The average weekly number of patient-provider visits and the relative percent difference in the average weekly number of visits between each 2020 pandemic periods and the baseline pre-pandemic periods are presented in Table 1. The *P*-values communicate the statistical significance of the decrease or increase in patient-provider visits for health equity determinant groups and for visit specialties relative to the total population. Overall, there was a 39% drop in patient-provider visits from pre-pandemic to the early 2020 pandemic period and a 24% drop from pre-pandemic to the late 2020 pandemic period. All health equity determinate groups and visit specialties showed drops from pre-pandemic to both 2020 pandemic periods, with visits to mental health specialty being the one exception. Mental health specialty showed a 20% increase in visits from pre-pandemic to early 2020 and a 33% increase late 2020. When compared to the total population, the relative percentage differences were statistically significant (*P* < .05) across all health equity determinate groups except white race (−39%, *P* = .081) and low poverty (−39%, *P* = .430) during the early 2020 pandemic period and ages 18-44 (−24%, *P* = .656), unknown ethnicity (−24%, *P* = .641), and low poverty (−24%, *P* = .366) during the late 2020 pandemic period. When compared to the total population, the differences in patient-provider visits from pre-pandemic to early and late 2020 pandemic periods were statistically significant for all specialties (*P* < .001).

**Figure 1.**
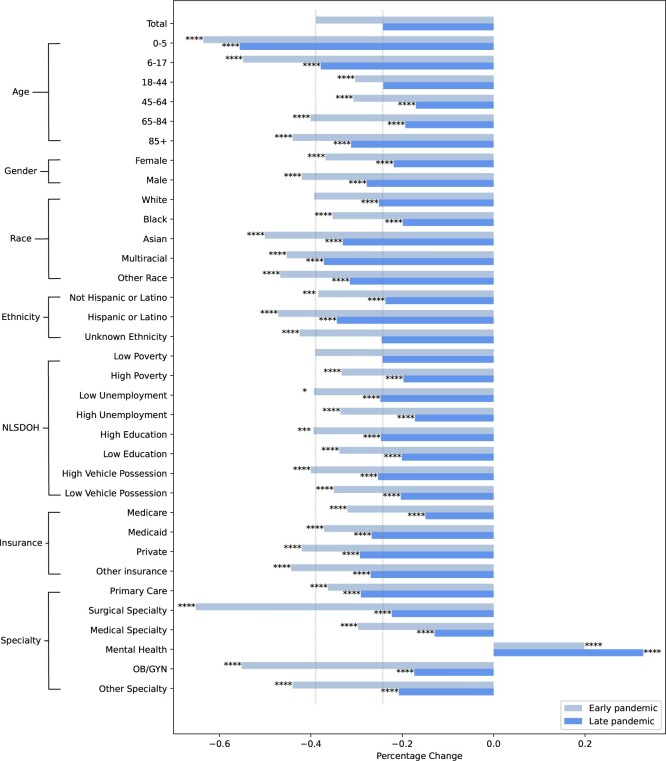
Changes in patient-provider visits by health equity determinant groups during the early and late pandemic periods. (1) Unknown, binary, other gender, and self-pay insurance status groups are not shown due to their small sample sizes. (2) The pale gray vertical line shows the percentage changes for the total group for ease of comparison. (3) Significance levels: *P-value < .05, **P-value < .01, ***P-value < .005, ****P-value < .001.

The health equity determinant groups that had extremely large drops (50% or more) in patient-provider visits from pre-pandemic to the early 2020 pandemic period included ages 0-5 (−64%), ages 6-17 (−55%), and Asian races (−50%). There were also extremely large relative differences in visits to surgical (−65%) and Obstetrics and Gynecology (OB/GYN) (−55%) specialties from pre-pandemic to early 2020. During the late 2020 pandemic period, ages 0-5 (−56%) was the only health equity determinant group with an extremely large drop in patient-provider visits from the pre-pandemic period. Figure 1 illustrates the changes in patient-provider visits by health equity determinant groups and visit specialties early and late 2020.

**Figure 2.**
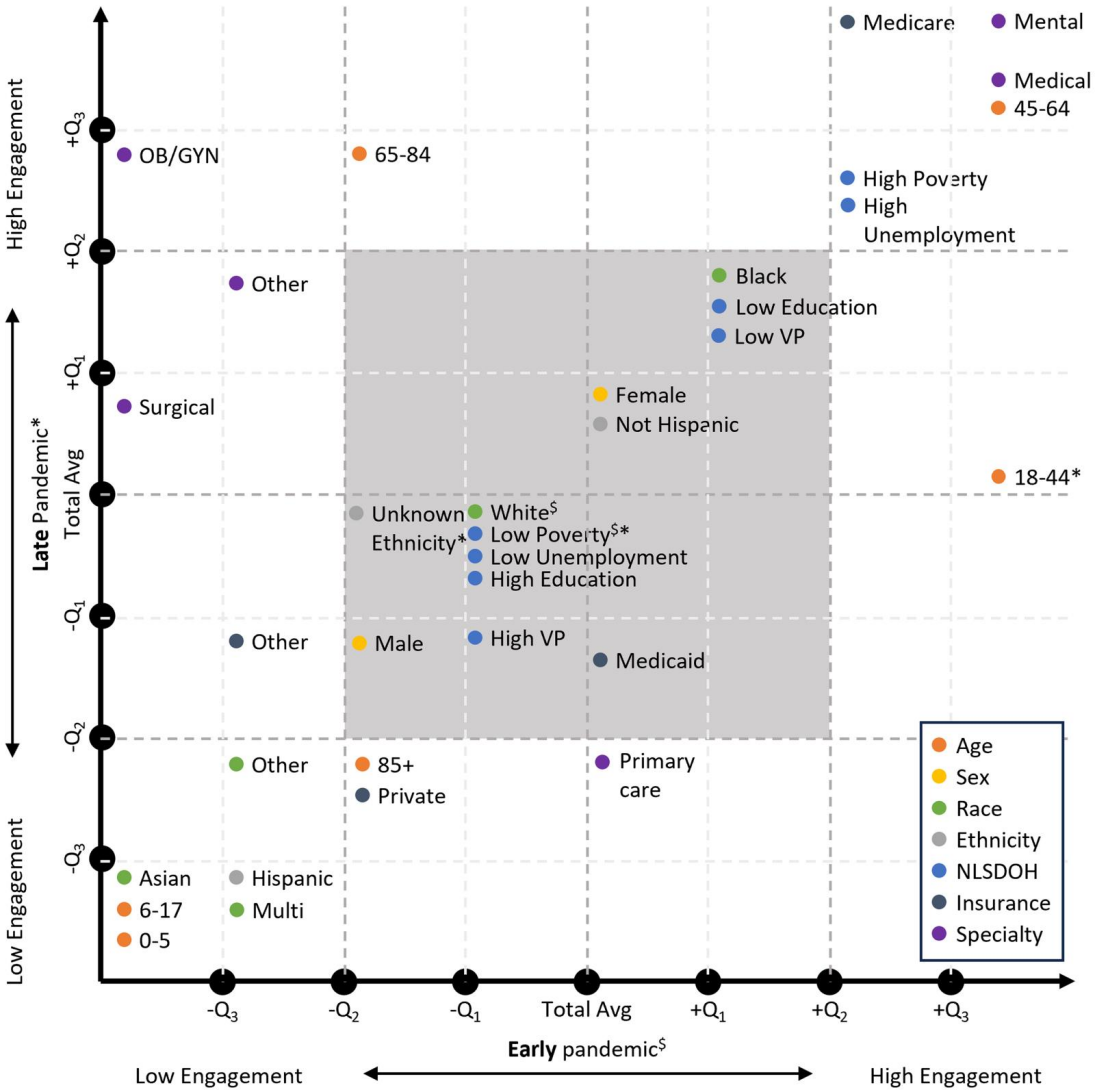
Illustration of the quantile locations of each group for the early and late pandemic periods. Higher quantile groups indicate higher patient-provider engagement and vice versa. (1) The point for each group was placed based on the quantiles, not at the exact location. (2) $ and * indicate that the change is not significantly different from the total group during the early and late pandemic period, respectively. (3) The square shaded gray refers to the area with −Q2 to +Q2 quantiles of change for both early and pandemic periods; groups in this area were not included in the generated patterns.

Figure 2 shows the quantile locations of each health equity determinant and visit specialty group for the early and late pandemic periods. Higher quantile groups indicate a smaller drop in patient-provider visits when compared to the total population during those periods, and lower quantile groups indicate a greater drop in visits. For the 21 groups falling outside the -Q_2_ to +Q_2_ quantile region, we discovered 4 patterns:

1. **Pattern 1: Groups with disproportionately smaller drops in patient-provider visits throughout the first year of the pandemic.** Health equity determinant groups: ages 45-64, high poverty, high unemployment, Medicare insurance. Visit specialty groups: mental health, medical specialty. When compared to the total population, these groups had smaller drops in visits throughout the first year of the pandemic (+Q2 or higher quantile for both early and late 2020 periods, *P* < .001).
2. **Pattern 2: Groups with disproportionately smaller drops in patient-provider visits early 2020 only.** Health equity determinant group: ages 65-84 groups. Visit specialty groups: OB/GYN and surgical specialty. When compared to the total population, these groups had smaller drops in visits in early 2020 only (−Q1 or lower quantile in early 2020 only, *P* < .001).
3. **Pattern 3: Groups with disproportionately greater drops in patient-provider visits throughout the first year of the pandemic.** Health equity determinant group: ages 0-5, ages 6-17, ages 85+, Asian race, Hispanic or Latino ethnicity, private insurance groups. When compared to the total population, these groups had greater drops in visits throughout the first year of the pandemic (−Q1 or lower quantile early 2020 and late 2020, *P* < .001).
4. **Pattern 4: Groups with a smaller drop in patient-provider visits early 2020 when compared to late 2020.** Health equity determinant group: ages 18-44 group. Visit specialty groups: primary care specialty. When compared to the total population, the age 18-44 group had a smaller drop in visits early 2020 (+Q3 quantile, *P* < .0001) and then had visit levels similar to the total population late 2020. Primary care visits were similar to the total population in early 2020 and then showed a smaller drop in visits late 2020 (−Q2 quantile, *P* < .001).

**Figure S1** shows the scatter plot of the changes in patient-provider visits by the groups during the early and late pandemic periods, with the identified patterns color-coded. Other, unknown, and multicategories were excluded from the discussion due to their difficulties in interpretation.

## Discussion

Our findings showed that most of the 34 health equity determinant and visit specialty groups, we considered showed statistically significant differences from the total population in terms of patient-provider visits between the pre-pandemic (*In-person Predominant*) period and the early 2020 pandemic (*Telehealth Predominant*) period or when in-person options returned in the late 2020 pandemic (*In-person & Telehealth*) period. We further described health equity determinant and visit specialty groups according to 4 distinct healthcare utilization patterns (Figure 1 and **Figure S1**). These patterns describe where disproportionately smaller or greater drops in visits occurred during the first year of the pandemic for certain health equity determinant and visit specialty groups. We focus our discussion on areas where these patterns provide insight into healthcare utilization for these groups during a changing landscape in telehealth offerings.

### Pattern 1: Groups with disproportionately smaller drops in patient-provider visits throughout the first year of the pandemic

There were 6 groups (ages 45-64, Medicare insurance, high poverty, high unemployment, mental health, medical specialty) that, relative to the total population, had smaller drops in patient-provider visits during both the early and late 2020 pandemic periods. Mental health specialty, in particular, had stark positive increases for both periods. This could be due to: first, the high mental health burden as a result of the degraded normal social support system and loneliness during isolation^32^; second, the high telehealth adoption of ∼90% for individual therapy across organizations that provide mental health services.^33^ Ages 45-64 may be one of the most tech-savvy and adaptive to change groups and, therefore, adapted well to the new modality. Contrary to most previous studies that observed lower telehealth access for people of lower socioeconomic status,^34^^,^^35^ groups of high poverty and high unemployment in our population showed smaller drops in healthcare utilization throughout the first year of the pandemic, including both the early 2020 period when we experienced a move to “telehealth predominant” care delivery and the late 2020 period when in-person options returned. It is worth ascertaining what approaches were taken to enable the more sustainable healthcare utilization experienced by groups. The reasons for more sustainable healthcare utilization for medical specialty and for Medicare insurance groups are unclear and warrant further investigation. Overall, groups with this pattern appear to show successful telehealth adoption and potentially more sustainable access to the benefits of telehealth.

### Pattern 2: Groups with disproportionately greater drops in patient-provider visits early 2020 only

Results indicated that the ages 65-84 group, OB/GYN specialty, and surgical specialty had greater drops in patient-provider visits for the “telehealth-predominant” period in early 2020, when compared to the total population. Once in-person options became available again late 2020, however, patient-provider visits returned to similar or even higher levels then what we saw in the total population. This result suggests that these groups may have experienced challenges to telehealth adoption or may have had a strong preference against telehealth options. The return to average when in-person visit options were returned also suggests that the greater drop in patient-provider visits may have been temporary. Others have observed low telehealth adoption for OB/GYN^36^ and surgical^37^ specialties during the pandemic, which was also true prior to the pandemic when compared to other specialties.^38^ The estimated greater drops in patient-provider visits to OB/GYN might also be due to the 33%-53% rate of prenatal checkup appointment cancellation or delay due to the pandemic as was also observed in other studies.^39^^,^^40^ Whereas greater drops in surgical specialty visits might be influenced by the announcement made by the Centers for Medicare & Medicaid Services (CMS) that all elective surgeries, non-essential medical, surgical, and dental procedures should be delayed.^41^ For health equity determinant groups, others have reported that older adults (65 years or older) are less likely to use telehealth when compared to their younger counterparts (less than 65 years old) due to a digital divide observed in older adults.^42^^,^^43^ After the estimated greater drop in visits early 2020 when compared to the total, we observed a smaller drop in visits for all 3 groups during the late 2020 pandemic period (Figure 2). This may indicate a compensation for a loss or delay in care once in-person options became available again. It is important to understand what challenges to telehealth adoption exist and what kinds of interventions can be implemented to minimize them in the event of a future pandemic requiring another widespread move to telehealth.

### Pattern 3: Groups with disproportionately greater drops in patient-provider visits throughout the first year of the pandemic

When compared to the total population, six groups had significantly greater drops in patient-provider visits during the early 2020 pandemic period, and greater drops remained for the late 2020 pandemic period after in-person options returned. Health equity determinate groups ages 0-5 and 6-17 had the greatest drops in visits pre-pandemic to the first year of the pandemic. While the cause for such apparent disengagement is unclear, the work of others offers some insight into potential telehealth utilization barriers among parents with pediatric patients.^44^^,^^45^ Some barriers for parents include fear of misdiagnosis, not being given the telehealth option, and not thinking telehealth could help meet the healthcare needs of their children. Asian race and Hispanic ethnicity groups also had greater drops in visits when compared to the total population, which aligns with previous studies highlighting that these groups are less likely to use telemedicine.^46^ Of the health equity determinant groups experiencing greater drops in visits compared to the total population throughout the first year of the pandemic, the ages 85+ and private insurance status groups had less drastic drops than the other groups (Figure 2). These groups also had lower visit rates compared to the total average during both periods (Table 1). The lower rates for those aged 85+ may in part be due to low digital literacy in this group,^42^^,^^43^ and the hesitancy of returning to the clinic in person as serious COVID complications are more frequently observed in this group.^47^ While reasons for possible disengagement among groups with private insurance are unclear, it is important to gain a better understanding of how members of this and other groups that are part of this pattern compensated for decreases in care in other ways, if at all.

### Pattern 4: Groups with a smaller drop in patient-provider visits early 2020 when compared to late 2020

It is helpful to understand how some groups may have benefited more from introducing telehealth when compared to the total population. The ages 18-44 group showed smaller drops in patient-provider visits during the early 2020 pandemic period when compared to the total population. However, once in-person options became available late 2020, this group returned to similar levels observed in the total population. A similar trend was estimated for primary care with visit levels similar to the total population early 2020, and then a greater drop in visits when compared to the total population late 2020. These findings suggest a potential benefit from telehealth options in the ages 18-44 group and for primary care. The observed trend for the ages 18-44 group might be influenced by high digital literacy among this group, and the trend seen in primary care may be due to relevance in the pandemic response for addressing patients’ general and COVID-19-specific health needs.^48^ The downward trend in visits, however, may suggest some temporary benefit or increase in needs, and such causes remain to be clarified. In particular, it is worth clarifying whether and how telehealth offerings during the first year of the pandemic may have contributed to the observed trend. For example, sustainable access to telehealth may be needed for groups that initially benefited early 2020 and then less so as fewer telehealth options were offered over time starting in late 2020.

Similar to the findings of others, our results demonstrate a drastic decrease in the number of in person visits early in the pandemic, which was accompanied by the rapid adoption of telehealth services.^13^^,^^49^^,^^50^ However, we estimated that the overall utilization of patient-provider visits did not fully recover to the pre-pandemic levels through the end of the study period, in contrast to the trend observed in several other studies.^13^^,^^49^ Our study provides additional evidence of the existence of health equity determinant groups being disproportionally affected by changing telehealth offerings during the pandemic. To our knowledge, this is the first study that identifies patterns of telehealth utilization during the first year of the pandemic. These patterns enabled identifying health equity determinant groups and visit specialties with disproportionately smaller or greater drops in patient-provider visits relative to the total population. While we have provided some plausible reasons for estimated differences with changing telehealth offerings during the first year of the pandemic, more work is needed to understand the observed dynamics in patient-provider visits. Furthermore, health equity determinants found by others to be associated with telehealth utilization that we did not study (eg, Internet access, English proficiency level, etc.),^11^^,^^14^^,^^51^ may be of interest to assess for differentially smaller or greater drops in patient-provider visits among sub-groups as we have in this study.

### Limitations and future directions

This study has several limitations. First, we leveraged our knowledge of telehealth offerings during certain time periods with very few in-person offerings early in the pandemic when stay-at-home orders were in place. While this enabled us to study patterns of healthcare utilization, the telehealth-predominant period may include some in-person visits. Furthermore, upon opening in-person visits, there may be other factors such as initial increases in wait times for appointments that influence our results. To control for such factors, a larger dataset that covers the years 2021 and 2022 would enable the use of washout periods between different telehealth offering time periods. Second, we did not distinguish between phone- and video-based telehealth in this study. Because of this, we are unable to study *what* telehealth offerings were adopted among health equity determinant groups during the periods under investigation. This is an area for further study given that the findings of others show differences in the use of phone-based and video-based telehealth by health equity determinants.^15^ Third, the analyzed SDOH measures were obtained at the census tract level, rather than at an individual level. Therefore, these measures define groups at the neighborhood level, and may not completely reflect the true status of an individual. Fourth, we cannot exclude the possibility that differences in healthcare utilization may just reflect actual differences in healthcare needs between populations. We were not able to identify whether a greater drop in patient-provider visits during pandemic periods indicates lower access to telehealth visits or lower healthcare needs during the pandemic. Fifth, this study was conducted with data from a cohort of JHM patients. Thus, while the results of our study may be applicable to Maryland residents, they may not generalize to other states. Last, patient-provider visits that occurred outside of JHM would not be part of our dataset, and thus undetectable. Nonetheless, strategies from this study may help to detect the unintended impacts of telehealth transformation at JHM. Understanding patterns of healthcare utilization following telehealth transformation is well-aligned with goals for the *JHM Telehealth Equity Dashboard*^52^ to support ongoing, system-wide assessment of how telehealth strategies may be affecting different patient populations in disparate ways. Further work is needed to leverage such a tool to monitor and detect problematic patterns of healthcare utilization among vulnerable populations in response to new digital health initiatives. Doing so can inform strategies to mitigate such unintended consequences.

## Conclusion

This work identified four main patterns of healthcare utilization during periods of changing telehealth offerings among health equity determinant and visit specialty groups. These patterns clearly identified groups that had disproportionally smaller or greater drops in visits during the first year of the pandemic, when compared to the total population. While this work did not specifically study vulnerable populations, the patterns of healthcare utilization we discovered set the stage for further studies of such groups. With emerging digital health transformation initiatives, including telehealth options, it is important to understand the potential to reduce or exacerbate disparities in access and quality of care. In addition, we should consider establishing safeguards for some groups to prevent the benefits of access to new digital health options being lost at future time periods.

## Supplementary Material

ooae093_Supplementary_Data

## Data Availability

The dataset cannot be shared publicly due to IRB restrictions on data obtained from participants without consent for sharing publicly.
