# Supplementary material for: Patterns of healthcare utilization according to health equity determinants during the first year of the pandemic at Johns Hopkins Medicine
Source: JAMIA Open. 2024 Oct 7;7(4):ooae093. doi: 10.1093/jamiaopen/ooae093 (PMC11458551; doi:10.1093/jamiaopen/ooae093)
Supplement: ooae093_Supplementary_Data [file ooae093_supplementary_data.docx]

**APPENDIX**

**Supplementary Table 1. List of encounter types.** The table lists the encounter types present in the dataset, along with their sttaus as a patient-provider visit.

| **Encounter type** | **Status** | **Category** |
| --- | --- | --- |
| Appointment | Included | Patient-provider visit (in-person, video, telephone) |
| Home visit | Included | Patient-provider visit (in-person, video, telephone) |
| Office visit | Included | Patient-provider visit (in-person, video, telephone) |
| Visit encounter | Included | Patient-provider visit (in-person, video, telephone) |
| Virtual visit | Included | Patient-provider visit (in-person, video, telephone) |
| History | Excluded | Not a patient-provider visit |
| Patient message | Excluded | Not a patient-provider visit |
| Scanned document | Excluded | Not a patient-provider visit |
| Refill | Excluded | Not a patient-provider visit |
| Telephone | Excluded | Not a patient-provider visit |
| Home care visit | Excluded | Not a patient-provider visit |
| Results only | Excluded | Not a patient-provider visit |
| Orders only | Excluded | Not a patient-provider visit |
| Anesthesia | Excluded | Not a patient-provider visit |
| Ancillary orders | Excluded | Not a patient-provider visit |
| Anti-coag visit | Excluded | Not a patient-provider visit |
| Ancillary procedure | Excluded | Not a patient-provider visit |
| Documentation | Excluded | Not a patient-provider visit |
| Patient outreach | Excluded | Not a patient-provider visit |
| Transcribe orders | Excluded | Not a patient-provider visit |
| Immunization | Excluded | Not a patient-provider visit |
| Allied health | Excluded | Not a patient-provider visit |
| Hospital encounter | Excluded | Not a patient-provider visit |
| Clinical support | Excluded | Not a patient-provider visit |
| E-consult | Excluded | Not a patient-provider visit |
| Procedure visit | Excluded | Not a patient-provider visit |
| Anesthesia event | Excluded | Not a patient-provider visit |
| Home care admission | Excluded | Not a patient-provider visit |
| Letter (out) | Excluded | Not a patient-provider visit |
| MyChart refill | Excluded | Not a patient-provider visit |
| Reconciled outside data | Excluded | Not a patient-provider visit |
| BPA | Excluded | Not a patient-provider visit |
| Erroneous telephone encounter | Excluded | Not a patient-provider visit |
| Abstract | Excluded | Not a patient-provider visit |
| Routine prenatal | Excluded | Not a patient-provider visit |
| Intake encounter | Excluded | Not a patient-provider visit |
| Home care referral | Excluded | Not a patient-provider visit |
| Infusion | Excluded | Not a patient-provider visit |
| Historical encounter | Excluded | Not a patient-provider visit |
| Erroneous encounter | Excluded | Not a patient-provider visit |
| Specialty pharmacy | Excluded | Not a patient-provider visit |
| Questionnaire series submission | Excluded | Not a patient-provider visit |
| Billing encounter | Excluded | Not a patient-provider visit |
| Prep for procedure | Excluded | Not a patient-provider visit |
| Recurring plan | Excluded | Not a patient-provider visit |
| Provider procedure | Excluded | Not a patient-provider visit |
| Education | Excluded | Not a patient-provider visit |
| Lab requisition | Excluded | Not a patient-provider visit |
| Committee review | Excluded | Not a patient-provider visit |
| Ophth exam | Excluded | Not a patient-provider visit |
| External contact | Excluded | Not a patient-provider visit |
| Evaluation | Excluded | Not a patient-provider visit |
| Follow-up | Excluded | Not a patient-provider visit |
| OB nurse history | Excluded | Not a patient-provider visit |
| Administratively closed | Excluded | Not a patient-provider visit |
| Postpartum visit | Excluded | Not a patient-provider visit |
| Post mortem documentation | Excluded | Not a patient-provider visit |
| Care plan review | Excluded | Not a patient-provider visit |
| Episode changes | Excluded | Not a patient-provider visit |
| Initial consult | Excluded | Not a patient-provider visit |
| Initial prenatal | Excluded | Not a patient-provider visit |
| EpicOnHand encounter | Excluded | Not a patient-provider visit |
| E-visit | Excluded | Not a patient-provider visit |
| Community orders | Excluded | Not a patient-provider visit |
| Nurse triage | Excluded | Not a patient-provider visit |
| Admission orders | Excluded | Not a patient-provider visit |
| External hospital admission | Excluded | Not a patient-provider visit |
| Consent form | Excluded | Not a patient-provider visit |
| Patient care review | Excluded | Not a patient-provider visit |
| Lactation consult | Excluded | Not a patient-provider visit |
| Empty | Excluded | Not a patient-provider visit |
| Affiliate encounter | Excluded | Not a patient-provider visit |
| E-consult community order | Excluded | Not a patient-provider visit |
| Multidisciplinary tumor board | Excluded | Not a patient-provider visit |
| E-medical second opinion | Excluded | Not a patient-provider visit |


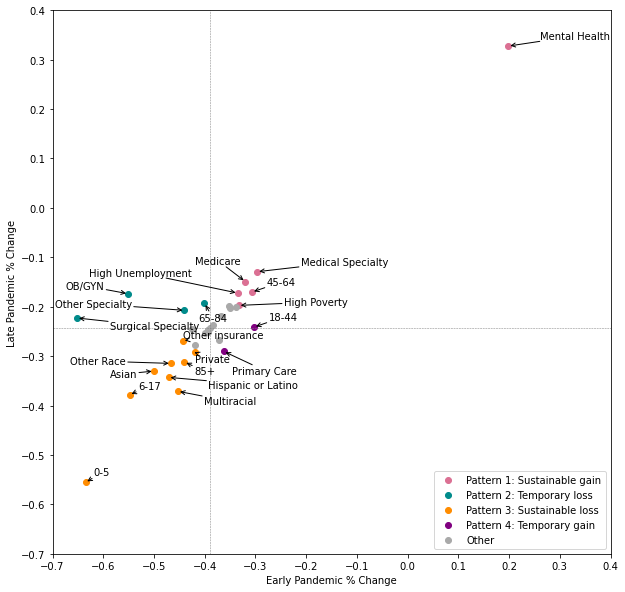


**Supplementary Figure 1.** **Scatter plot of the changes in patient-provider visits by the groups during the early and late pandemic periods.** Note: The grey vertical and horizontal lines show the percentage changes for the total group for ease of comparison
